# Driving arginine catabolism to activate systemic autophagy

**DOI:** 10.1080/27694127.2022.2040763

**Published:** 2022-03-24

**Authors:** Yiming Zhang, Brian N. Finck, Brian J. DeBosch

**Affiliations:** a Department of Pediatrics, Washington University School of Medicine, St. Louis, MO 63110; b Department of Medicine, Washington University School of Medicine, St. Louis, MO 63110; c Department of Cell Biology & Physiology, Washington University School of Medicine, St. Louis, MO 63110

## Abstract

Macroautophagy/autophagy is a conserved cellular self-digestive mechanism to catabolize superfluous or damaged cellular components to maintain cell homeostasis. Impaired autophagy underlies multiple pathophysiological states, including aging, neurodegenerative, inflammatory, and metabolic diseases. Intermittent fasting and caloric restriction are effective means by which to activate autophagy, yet relatively few people can sustain such intensive interventions in real-world settings. Moreover, current pharmacotherapies do not yet fully exploit autophagic flux as a target mechanism. Here, we discuss recent work, which demonstrates that arginine catabolism is a tractable process to activate autophagy with utility to treat obesity and its complications. Hepatocyte-specific transgenic activation of arginine catabolism, or systemic administration of an anti-tumor pharmacotherapy, pegylated arginine deiminase, each promote energy expenditure and insulin sensitivity, and reduce dyslipidemia and hepatic steatosis in obese mice. These effects depend upon hepatocyte *Fgf21*, and whole-body *Becn1* expression. The data suggest that hepatocyte and systemic arginine catabolism drive autophagy, and identify an index pharmacological agent to leverage this process.

Autophagy is a homeostatic mechanism conserved across species. Whereas autophagic deficiency is itself deleterious to the organism, coordinated autophagic activation is already an important and widely recognized clinical practice. This typically manifests as intermittent fasting or caloric restriction – lifestyle interventions that are difficult to sustain in real-world practice. Moreover, several existing pharmacotherapies are also likely to exert at least part of their effects by stimulating autophagic flux, although the mechanistic actions and full applicability of these agents across diseases remain underappreciated.

We initially defined the arginine ureahydrolase, ARG2, as a fasting-induced hepatocyte factor. Similar to the canonical urea cycle enzyme, ARG1, ARG2 also hydrolyzes arginine to generate ornithine and urea. Yet hepatic ARG2, but not ARG1, is uniquely induced during fasting and after glucose uptake blockade in hepatocytes. Whereas the precise purpose of specific, dynamic regulation of *Arg2* expression is not yet clear, hepatocyte-specific *Arg2* overexpression increases energy expenditure, blocks hepatocyte inflammation and improves glucose homeostasis in diet-induced and genetically obese animals. These findings prompted us to hypothesize that activating hepatocyte arginine catabolism *per se* is sufficient to drive metabolic improvements.

We therefore sought to separate the effect of arginine hydrolysis from multiple other potential ARG2-specific functions in augmenting energy homeostasis. This included any potential binding, scaffolding, and signal transduction effects, synthesis of ornithine and urea, or mitochondrial localization-specific functions of transgenic *Arg2* expression. To that end, we overexpressed a structurally distinct arginine iminohydrolase, arginine deiminase (ADI, encoded by the *arcA* gene) in a hepatocyte-specific manner [1]. *arcA* is a bacterial virulence factor that cleaves arginine to citrulline and ammonia. The net effect of this action is to evade host inflammatory responses by shunting arginine away from host NOS2 (nitric oxide synthase 2, inducible). Accordingly, *arcA* overexpression reduces hepatocyte inflammatory gene expression in genetically diabetic db/db mice when compared with GFP-overexpressing controls. Moreover, *arcA* overexpression also phenocopies the metabolic *Arg2-*overexpression phenotype in *db/db* mice. Specifically, *arcA* attenuates weight gain, increases caloric expenditure, reduces hepatic steatosis, and improves glucose tolerance. We also observed increased intrahepatic accumulation of the autophagy marker protein LC3B-II, and increased circulating levels of the fasting-induced hepatokine, FGF21 (fibroblast growth factor 21), in *arcA*-overexpressing mice when compared with GFP-overexpressing mice. Together, the data indicate that hepatocyte arginine catabolism *per se* enhances whole-body energy homeostasis in obesity. These changes associate with known fasting-like signals; increased autophagic flux and FGF21 secretion.

We then explored the translational potential of arginine targeting by quantifying the metabolic effects of PEGylated arginine deiminase (ADI-PEG20). ADI-PEG20 is a stabilized, systemically-administered enzyme that is currently under investigation as an anti-tumor agent against hepatocellular carcinoma and other solid tumors. Like *arcA*, ADI-PEG20 also converts arginine to citrulline and ammonia, thereby diverting arginine from proliferating cells. Five-week ADI-PEG20 treatment recapitulates hepatocyte *Arg2*- and *arcA*-induced improvements in body fat and calorie expenditure, insulin sensitivity, glucose tolerance, and peripheral and intrahepatic fat content. These improvements again associate with increased liver expression of *Fgf21* and circulating FGF21 concentrations, and hepatic LC3B-II abundance. Next, we determined whether hepatocyte-specific deletion of *Fgf21* or *Becn1*, which encodes a component of the autophagy phosphatidylinositol 3-kinase complex, reverses the metabolic effects of ADI-PEG20 in a Western diet-induced obese mouse model. Hepatocyte-specific *fgf21* and *becn1* deletion both partly reverse ADI-PEG20 effects on body fat, serum lipids and hepatic triglyceride accumulation. However, both genes are dispensable for ADI-PEG20-mediated improvements in glucose tolerance. In contrast, whole-body *Becn1* haplo-insufficiency reverses ADI-PEG20-mediated reductions in fasting glucose in addition to blocking the effects on insulin tolerance and dyslipidemia. Moreover, single-cell ATAC sequencing revealed broad effects of on the accessible chromatin landscape in the hepatocyte population, which are abrogated in *Becn1* haplo-insufficient mice. Together, these data indicate that systemically administered ADI-PEG20 exerts pleiotropic hepatocyte-specific and systemic effects that require fasting-like signals through FGF21 and BECN1. These complementary mechanisms mediate improvements in glucose, lipid, and insulin homeostasis downstream of arginine catabolism (Figure 1).

**Figure 1.**
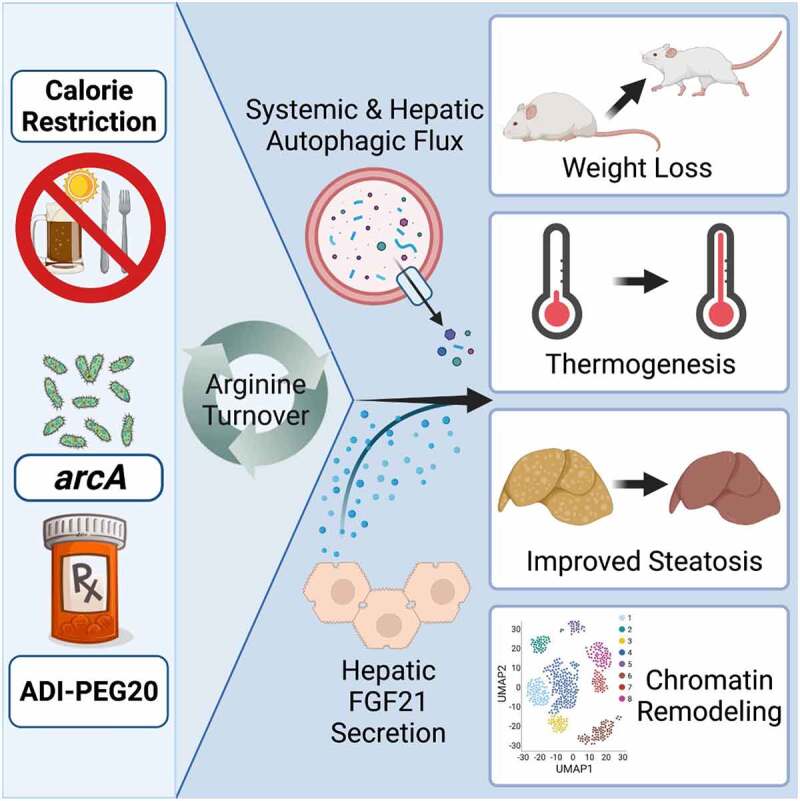
Schematic describing multiple inputs through which to activate arginine turnover. Arginine catabolism drives hepatic and systemic autophagy and hepatokine secretion. This ultimately yields multi-systemic metabolic improvements.

Autophagy activation is an emerging and promising treatment strategy across aging, proteostatic, inflammatory, neurodegenerative, and metabolic diseases. Important barriers to fully advance this strategy remain an incomplete understanding of autophagy-stimulating pathways that are amenable to pharmacotherapy, and complete mechanistic insight into pharmacotherapies that activate autophagy. Using obese, diabetic mouse models as a paradigm of its broader utility, we nominate hepatocyte- and systemic arginine catabolism as targetable, autophagy-promoting processes. We extend this to demonstrate that ADI-PEG20 leverages these processes to abate obesity and its complications. These findings warrant a more detailed examination of hepatocellular effects of altered arginine flux, and detailed mechanisms by which this regulates autophagy. Moreover, we anticipate that advancing ADI-PEG20 or perhaps other arginine-based therapies clinically will augment our armamentarium of agents to deploy across the spectrum of diseases with pathophysiology enrooted in autophagy deficiency.
